# Effect of menstrual cycle and contraceptive pill phase on aspects of exercise physiology and athletic performance in female athletes: protocol for the *Feminae* international multisite innovative project

**DOI:** 10.1136/bmjsem-2023-001814

**Published:** 2023-11-24

**Authors:** Kirsty J Elliott Sale, Tessa R Flood, Shawn M Arent, Eimear Dolan, Bryan Saunders, Mette Hansen, Johanna K Ihalainen, Ritva S Mikkonen, Clare Minahan, Jane S Thornton, Kathryn E Ackerman, Constance M Lebrun, Craig Sale, Trent Stellingwerff, Paul A Swinton, Anthony C Hackney

**Affiliations:** 1 Department of Sport and Exercise Sciences, Manchester Metropolitan University Institute of Sport, Manchester, Manchester, UK; 2 Department of Exercise Science, Arnold School of Public Health, University of South Carolina, Columbia, SC, USA; 3 Applied Physiology and Nutrition Research Group, - Center of Lifestyle Medicine, Faculdade de Medicina FMUSP, Universidade de São Paulo, Sao Paulo, Brazil; 4 Department for Public Health, Aarhus University, Aarhus, Denmark; 5 Faculty of Sport and Health Sciences, Finland / Finnish Institute of High Performance Sport KIHU, University of Jyväskylä, Jyvaskyla, Finland; 6 Sports Technology Unit, Faculty of Sport and Health Sciences, University of Jyväskylä, Vuokatti, Finland; 7 Griffith Sports Science, Griffith Health, Griffith University, Gold Coast, Queensland, Australia; 8 Australian Institute of Sport, Australian Sports Commission, Canberra, ACT, Australia; 9 Western Centre for Public Health & Family Medicine, Schulich School of Medicine & Dentistry, Western University, London, Ontario, Canada; 10 Wu Tsai Female Athlete Program, Division of Sports Medicine, Boston Children's Hospital, Boston, MA, USA; 11 Neuroendocrine Unit, Massachusetts General Hospital and Harvard Medical School, Boston, MA, USA; 12 Department of Family Medicine, Faculty of Medicine & Dentistry, University of Alberta, Edmonton, Alberta, Canada; 13 Canadian Sport Institute Pacific, Victoria, British Columbia, Canada; 14 School of Health Sciences, Robert Gordon University, Aberdeen, UK; 15 Department of Exercise & Sport Science – Department of Nutrition, The University of North Carolina at Chapel Hill, Chapel Hill, NC, USA

**Keywords:** Female, Physiology, Performance

## Abstract

The idiom *‘more high-quality research is needed*’ has become the slogan for sport and exercise physiology-based research in female athletes. However, in most instances, it is challenging to address this gap of high-quality research in elite female athletes at a single study site due to challenges in recruiting enough participants with numerous menstrual cycle and contraceptive pill permutations. Accordingly, we have assembled an international multisite team to undertake an innovative project for female athletes, which investigates the effects of changes in endogenous and exogenous oestrogen and progesterone/progestins across the menstrual cycle and in response to second-generation combined monophasic contraceptive pill use, on aspects of exercise physiology and athletic performance. This project will employ the current gold-standard methodologies in this area, resulting in an adequately powered dataset. This protocol paper describes the consortium-based approach we will undertake during this study.

**WHAT IS ALREADY KNOWN ON THIS TOPIC**

- At present, there is insufficient high-quality data to determine the effects of menstrual cycle phase and contraceptive pill use on athletic performance in female athletes. This lack of credible information limits our ability to guide female athletes in relation to fluctuations in ovarian hormones either across the menstrual cycle or in response to contraceptive pill use.

**WHAT THIS STUDY ADDS**

- This innovative, multisite project will provide novel, high-quality data, with study components adequately powered to test specific hypotheses relevant to female athletes.

**HOW THIS STUDY MIGHT AFFECT RESEARCH, PRACTICE OR POLICY**

- This project will allow us to provide guidance to female athletes (ie, changing or confirming practice) about the effects of two of the most experienced hormonal profiles on aspects of physiology and athletic performance. In addition, this project will assess a research design blueprint for use in future studies.

## Introduction

At present, there are insufficient high-quality data to determine if (1) the cyclical fluctuations in endogenous oestrogen and progesterone across the menstrual cycle (MC) and (2) the downregulation of endogenous oestrogen and progesterone with simultaneous administration of exogenous oestrogen and progestins from combined, monophasic contraceptive pill (COCP) use affects female exercise physiology and athletic performance.^1–3^ Specifically, only 53 studies investigating the effects of MC phase on exercise performance were identified, and just four of these were classified as high quality.^1^ Similarly, only 30 studies examining the effects of oral contraceptives on exercise performance were identified, and just five were classified as high quality.^2^ In 2021, Elliott-Sale *et al*^3^ produced a statement on methodological considerations that provided recommendations for standardised, high quality (ie, best practices) research design for studies investigating the effects of ovarian hormone profiles on outcomes related to physiology and athletic performance. Such research is needed to establish a cause-and-effect relationship for any differences in exercise physiology and athletic performance between the different phases of the MC, phases of COCP use, and women using or not using COCPs. The impact of natural (ie, oestrogen and progesterone) and synthetic (ie, oestrogen and progestin) hormones on women’s exercise capacity is important for active and elite female athletes to optimise their training schedules and athletic performance.

We have previously described the concept of the *Feminae* consortium.^4^ In brief, we have assembled a multisite team, supported by a scientific steering group and an athlete advisory group, to address many of the pitfalls associated with current female athlete-based sports science research, namely poor-quality, underpowered studies. This multicentre, adequately powered project will investigate the effects of circulating fluctuations in endogenous and exogenous oestradiol and progesterone/progestins experienced during the MC and from COCP use on aspects of exercise physiology and athletic performance in female athletes. This project is an innovative, international venture involving seven sites worldwide which will employ the same research design in two populations (ie, eumenorrheic (EUM) women and COCP users).^3^ The combined data across sites will be analysed and interpreted, producing an adequately powered dataset. In addition, we aim to establish a research design blueprint that can be used to assess other outcomes in future studies of female athletes.

## Methods and analysis

Disclosure: this project will be conducted with cis-gender women (ie, an adult who was assigned female at birth and whose gender identity is female). For this project, “female” will be used as an adjective and “woman/women” as a noun (singular/plural, meaning an adult female human being).

### Research design

**Figure 1:**
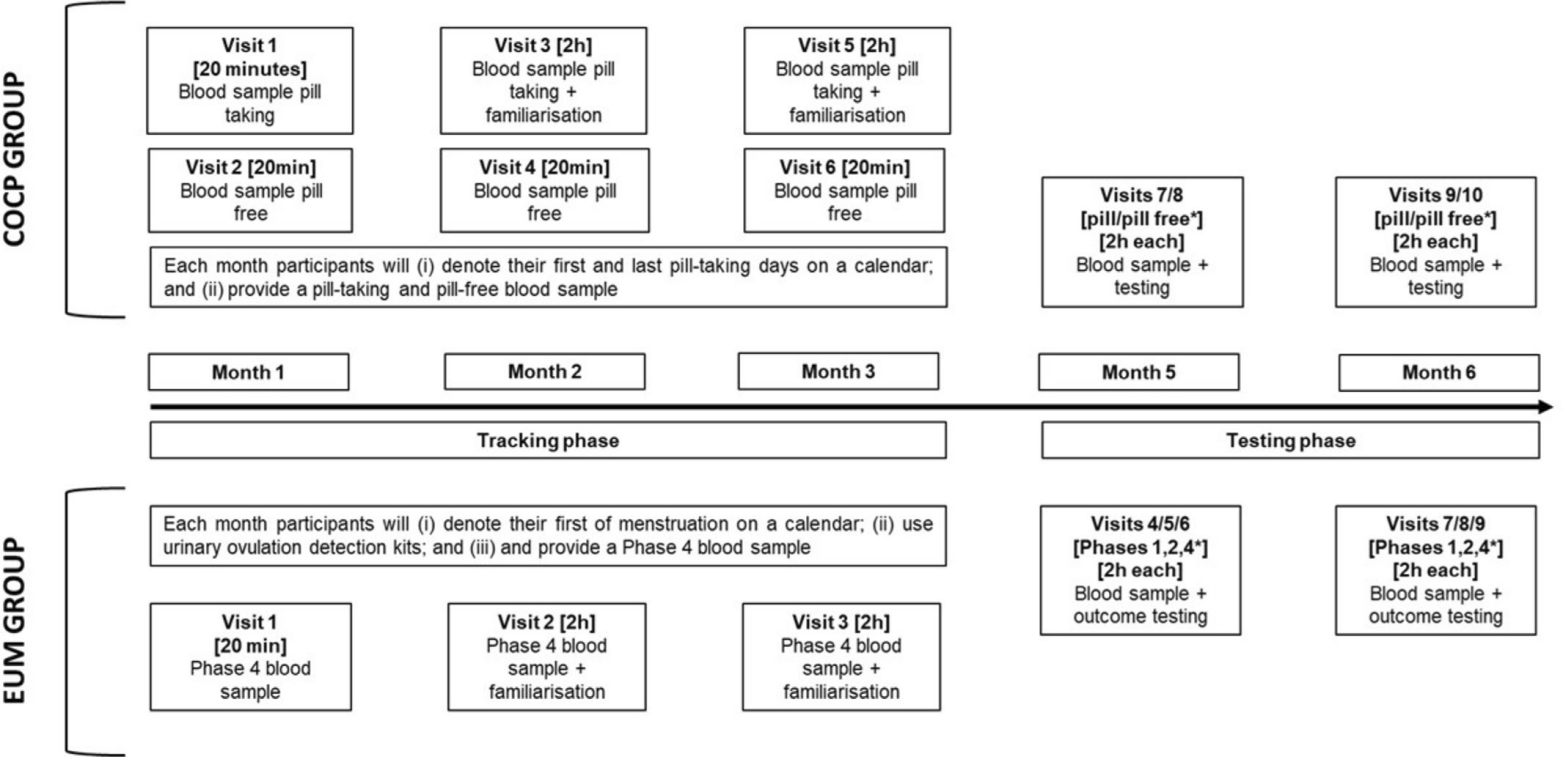
Study timeline. Note that the months do not have to be consecutive. *Denotes that these phases will be assigned in a random order.

This project will employ an observational, random testing order, single-blinded, repeated measures approach. Figure 1 shows the study timeline and corresponding hormonal profiles. A random testing order will be employed, meaning that phase 1, 2 and 4 (table 1; adapted from Elliott-Sale *et al*^3^) testing in the EUM group and pill-taking and pill-free testing in the COCP group will not be completed in sequence. A single-blind research design will be employed; although participants cannot be blinded, the experimenter will be blind to the intended testing time point. Experimenter blinding will be achieved by having an independent party schedule the testing session based on the participant’s hormonal profile without disclosing this to the experimenter who will conduct the testing session. On completion of the study, the blinded experimenters will be asked if blinding was achieved and maintained. To reduce participants’ preconceptions about the effects of the menstrual or COCP cycle phase on the study outcomes, participants will receive a standardised summary of this research area (box 1), intended to neutralise any placebo or nocebo effects. Following data collection (ie, on study completion), participants will answer several exit questions relating to their prestudy expectations and MC/COCP tracking behaviours (box 2).

Box 1 **Information participants will receive about previous research on and experiences with the menstrual and contraceptive pill cycles and athletic performance**

**Your experiences**

We accept and understand that every woman’s body is different and that every woman’s experience of the menstrual cycle or taking oral contraceptives is different. Moreover, we acknowledge that, for some but not all of you, your menstrual cycle or contraceptive pill use affects your sports/exercise training and performance. These perceptions and experiences are yours and are unique to you.

**The research evidence**

How the menstrual cycle or contraceptive pill use affects sport/exercise training and performance is a hot topic right now. You might have seen/heard a lot of talk about this topic in the media and especially on social media. All the researchers involved in this study have extensive experience and knowledge of the research evidence on this topic. Right now, the scientific community has not reached an agreement about if and how the menstrual cycle and contraceptive pill use affect athletic training or performance.

**What we need from you—‘Impartiality’**

We are asking you to forget what you have seen and heard with regards to the effects of the menstrual cycle and contraceptive pill use on athletic training and performance. In other words, keep an open mind during this study. Do not try to predict or anticipate a certain response or outcome during this study. Try not to let social media, other people, or research papers influence your opinions while taking part in this study. We want the findings from this study to be true and accurate and not tainted by any existing views.

Let us know if you have any questions about what you have read here.

Box 2 **Questions participants will be asked after they complete the study**

1. Prior to enrolment in this study, did you track your menstrual cycle or contraceptive pill use?
2. If yes, how did you track this information?
3. Prior to taking part in this study, did you believe that the menstrual cycle or contraceptive pill use affected female athlete performance?
4. Prior to taking part in this study, did you believe that the menstrual cycle or contraceptive pill use affected your performance?
5. If yes to question 3 or 4, was this belief based on our own personal experience or something you read/heard?
6. Can you provide details on either your lived experience or what you read/heard?

**Table 1:** Eumenorrheic phase descriptors (adapted from Elliott-Sale *et al*^3^)

Phase 1	Indicated by the onset of bleeding until day 5 Oestrogen and progesterone levels are low
Phase 2	Oestrogen is higher than during phases 1 and 4 Progesterone higher than during phase 1 but lower than 6.36 nmol/L Must be followed (within 48 hours) by a positive luteinising hormone surge
Phase 4	+ 6, 7, or 8 days after ovulation has been confirmed Oestrogen is higher than phase 1 but lower than phase 2 Progesterone > 16 nmol/L

### Participants

Participants will be cis-gender women aged ≥ 18 and ≤ 40 years, with a body mass index ≥ 19.5 and ≤ 25 kg∙m^2^; an exception can be made if an athlete with a BMI > 25 kg∙m^2^ is considered to have mesomorphic somatotype traits (ie, shoulders wider than the waist, developed athletic musculature). Participants will be defined as healthy, that is, display no contraindications to the proposed exercise testing established from a health screening questionnaire, verbal enquiry and consent forms. In addition, participants will be excluded if they report having an injury that could be worsened by exercise, have any medical condition or are taking any medication known to affect the outcome measures. Examples include diagnosed hyperandrogenism from polycystic ovary syndrome, congenital adrenal hyperplasia, or other differences in sexual development. Participants will not be smokers. Participants will provide gravidity and parity details, must not be within 12 months of childbirth, and must not be currently breastfeeding or pregnant. Participants will be recruited based on their self-reported endogenous hormonal profile (ie, EUM or COCP users). Participants' training status will be classified using the criteria outlined by McKay *et al*^5^ and must be tier 2 athletes or above. In addition, participants will be considered based on relative maximal oxygen consumption^6^; they must be performance level 2 or above (ie, > 37 mL/kg/min). Participants will be considered eligible if they fit either one of these criteria. All participants will receive a participant information sheet and have at least 24 hours between receiving it and signing the informed consent.

#### Sample size

Each site will aim to recruit and test a minimum of 10 EUM and 10 COCP users, resulting in an expected total of 70 EUM participants and 70 COCP users in the final combined dataset. The group size was based on a balance between pragmatic considerations of the recruitment capacity across the different sites and a range of simulations of mixed effects models with random effects (participants and sites) and small overall effect sizes (eg, hedges *g* of 0.1–0.2) likely to generate adequate power (1-beta~0.8).

#### EUM group

Participants will: (1) have MC lengths ≥ 21 days and ≤ 35 days resulting in nine or more consecutive periods per year; (2) provide evidence of a luteinising hormone surge; (3) demonstrate the required hormonal profile as defined by Elliott-Sale *et al*^3^ ; and (4) have not used any type of hormonal contraceptive for a minimum of 3 months, but ideally 6 months, before enrolment. MC phase tracking (figure 1) will be achieved by (1) denoting the first and last day of menstruation on a calendar for each cycle; (2) undertaking urinary ovulation detection using kits (visual confirmation will be provided to the researcher); and (3) providing a phase 4 blood sample. A posteriori exclusion will be applied to participants not meeting the stipulated hormonal profiles. Two of the ‘tracking phase’ visits will be used as familiarisation sessions or two separate familiarisation sessions will be scheduled at times convenient for the participants, in addition to the mandated tracking phases session where blood samples need to be provided. EUM participants will be tested during phases 1, 2 and 4 of their cycle.

#### Combined, monophasic contraceptive pill (COCP) group

Participants must have been taking their COCP ≥ 3 months before recruitment and must be taking a combined, monophasic, second-generation COCP (see table 2 for permitted brands).

**Table 2:** Oral contraceptives acceptable for inclusion in the study

Country	Brand name	Oestrogen content	Progestin content
UK	Levest	Ethinylestradiol 30 μg	Levonorgestrel 150 μg
	Microgynon 30	Ethinylestradiol 30 μg	Levonorgestrel 150 μg
	Ovranette	Ethinylestradiol 30 μg	Levonorgestrel 150 μg
	Rigevidon	Ethinylestradiol 30 μg	Levonorgestrel 150 μg
	Elevin	Ethinylestradiol 30 μg	Levonorgestrel 150 μg
	Maexeni	Ethinylestradiol 30 μg	Levonorgestrel 150 μg
Australia	Femme-Tab ED 30/150	Ethinylestradiol 30 μg	Levonorgestrel 150 μg
	Levlen ED	Ethinylestradiol 30 μg	Levonorgestrel 150 μg
	Microgynon 30 ED	Ethinylestradiol 30 μg	Levonorgestrel 150 μg
	Monofeme	Ethinylestradiol 30 μg	Levonorgestrel 150 μg
	Nordette	Ethinylestradiol 30 μg	Levonorgestrel 150 μg
	Evelyn 150/30 ED	Ethinylestradiol 30 μg	Levonorgestrel 150 μg
	Eleanor 150/30 ED	Ethinylestradiol 30 μg	Levonorgestrel 150 μg
	Micronelle 30 ED	Ethinylestradiol 30 μg	Levonorgestrel 150 μg
Canada	Alesse	Ethinylestradiol 30 μg	Levonorgestrel 100 μg
	Min-Ovral	Ethinylestradiol 30 μg	Levonorgestrel 150 μg
Brazil	Ciclo 21, Microvlar	Ethinylestradiol 30 μg	Levonorgestrel 150 μg
Finland	Levesia	Etinylestradiol 20 μg	Levonorgestrel 100 μg
	Microgynon	Etinylestradiol 30 μg	Levonorgestrel 150 μg
USA	Alesse	Ethinylestradiol 20 μg	Levonorgestrel 100 μg
	Altavera	Ethinylestradiol 30 μg	Levonorgestrel 150 μg
	Amenthia	Ethinylestradiol 30 μg	Levonorgestrel 150 μg
	Aviane	Ethinylestradiol 20 μg	Levonorgestrel 100 μg
	Camrese	Ethinylestradiol 30 μg	Levonorgestrel 150 μg
	Introvale	Ethinylestradiol 30 μg	Levonorgestrel 150 μg
	Jolessa	Ethinylestradiol 30 μg	Levonorgestrel 150 μg
	Lessina	Ethinylestradiol 20 μg	Levonorgestrel 100 μg
	Levora	Ethinylestradiol 30 μg	Levonorgestrel 150 μg
	Daysee	Ethinylestradiol 30 μg	Levonorgestrel 150 μg
Denmark	Anastrella	Ethinylestradiol 30 μg	Levonorgestrel 150 μg
	Femicept	Ethinylestradiol 30 μg	Levonorgestrel 150 μg
	Leverette	Ethinylestradiol 30 μg	Levonorgestrel 150 μg
	Microgyn	Ethinylestradiol 30 μg	Levonorgestrel 150 μg
	Microstad	Ethinylestradiol 30 μg	Levonorgestrel 150 μg
	Rigevidon	Ethinylestradiol 30 μg	Levonorgestrel 150 μg

ED, every day formulation.

Phase tracking (figure 1) will be achieved by (1) denoting the first and last pill-taking days on a calendar and (2) providing a pill-taking (days 18–20) and pill-free (days 5–7) blood sample. A posteriori exclusion will be applied to data from testing time points that do not comply with the stipulated hormonal profiles. Two of the ‘tracking phase’ visits will be used as familiarisation sessions. COCP users will be tested once during the pill-taking phase (any day between days 14 and 21) and once (days 4, 5, 6, or 7) during the pill-free phase.

### Consistency measures

Time of day (ie, ± 2 hours of the first testing session), prior exercise (ie, none or minimal within the 48 hours before testing; in the case of minimal exercise, the activities must be replicated for each testing session), caffeine ingestion (ie, none within 12 hours before testing), dietary intake (ie, dietary replication in the 24 hours before each testing session; diet will be recorded by the participants using either photographs on a phone/camera or noted using pen and paper or equivalent), nutritional supplementation (ie, same supplementation throughout the programme timeframe) and alcohol consumption (ie, none within the 24 hours before testing) will be standardised or replicated throughout the ‘testing phase’ as these have been shown to affect both the concentration of reproductive hormones as well as performance outcomes.^7–9^ Participants will be asked to complete a training and diet questionnaire at the start of the study (online supplemental file 1) Participants will be asked a series of questions (table 3) related to training, diet and wellness factors at the start of each testing session to establish the consistency of these factors.

10.1136/bmjsem-2023-001814.supp1 Supplementary data

**Table 3:** Standard questions to be used at the start of each laboratory visit

	Training-related questions
Question 1	Have you made significant/substantial changes (ie, doing more or less) to your training/exercise/sport programme/schedule?
Question 2	If yes, please briefly describe the changes made to your routine and the new regime adopted? (be as specific as possible)
Question 3	Is there anything else, training-related, that you would like to share with us, which you feel might impact/affect your participation in this study?
	**Dietary-related questions**
Question 1	Have you made significant/substantial changes (ie, eating more or less or adding something new) to your diet?
Question 2	If yes, please briefly describe the changes made to your diet and the new diet adopted? (be as specific as possible)
Question 3	Is there anything else, diet-related, that you would like to share with us, which you feel might impact/affect your participation in this study?
	**Perceived wellness questions**
Question 1	How do feel physically(ie, your body)today? Answer (please circle): Excellent good neutral bad terrible
Question 2	Do you have any physical symptoms you would like to make us aware of? Answer (please write down symptoms):
Question 3	How do feel emotionally(ie, your feelings/mood)today? Answer (please circle): Excellent good neutral bad terrible
Question 4	Do you have any emotional symptoms you would like to make us aware of? Answer (please write down symptoms):

### Testing sessions

Each testing session will follow the same order, lasting < 2 hours: blood sampling, assessment of height and weight, a standardised warm-up and stretching routine, characteristic outcome measurements and a bespoke cycling protocol (parts 1 and 2), incorporating a standardised cool-down period. The characteristic outcome measurements will be performed in the following order: balance, push-ups, agility, handgrip, countermovement jump, and flexibility.

#### Venous blood sample

A trained phlebotomist will draw a blood sample from an antecubital vein. Approximately 10 mL will be collected into a serum tube. Serum samples will be left to clot for 30 min before being centrifuged at room temperature (~20°C). Samples will be centrifuged at 3000 g for 10 min and then stored in a −80°C freezer for the subsequent determination of endogenous 17 β-oestradiol and progesterone. Samples will be transported between laboratories on −20°C dry-ice or in a −80°C freezer and will only be subjected to one freeze–thaw cycle.

#### Height and body mass

Using a stadiometer, height will be measured in bare feet, with minimal clothing and no impeding hairstyles. Participants will be positioned in the ‘Frankfort Plane’ and asked to take a deep breath, which they hold, while the measurement is taken. Height will be measured at the first laboratory visit only. Body mass will be measured at every testing session by an experimenter using the same calibrated scales on each occasion (ie, within and between participants), and the participants will wear minimal clothing and no shoes.

#### Standardised warm-up and stretching

Participants will complete a 5 min, 15–100 W (self-selected, but recorded and replicated for each subsequent testing session) warm-up on a cycle ergometer. Participants will then undertake the following warm-up stretching exercises, which will be led/supervised by an experimenter: modified Hurdler’s stretch, kneeling stretch, supine hamstrings stretch, overhead triceps stretch, seated bent-knee biceps stretch and child’s pose.

#### Balance: stork balance stand test

In bare feet, participants will place their hands on their hips and then position their non-supporting foot against the inside knee of the supporting leg. They will be given 1 min to practice the balance. They must raise their heel to balance on the ball of the foot. The stopwatch will be started as the heel is raised from the floor. The stopwatch will be stopped if any of the following occurs: (1) the hand(s) come off the hips; (2) the supporting foot swivels or moves (hops) in any direction; (3) the non-supporting foot loses contact with the knee; (4) the heel of the supporting foot touches the floor. The total time in seconds will be recorded. One minute’s rest will be allowed between attempts. Three attempts using the same supporting leg will be offered. However, if improvements are made, the participants may continue to perform the test beyond three attempts. The outcome measure is the longest total time of a single test recorded in seconds. Bouteraa *et al*^10^ reported an intraclass correlation coefficient (ICC) of 0.90 for repeated stork static balance test trials in female basketball players.

#### Muscular endurance: push-ups

The push-up test will be administered with participants starting in a modified knee push-up position (legs together, lower leg in contact with the mat, back straight, hands shoulder-width apart, head up, using the knees as the pivotal point). The participants will raise their body by straightening their elbows and returning to the ‘down’ position until their chin or chest touches the mat. Their stomach should not touch the mat. The participant’s back must remain straight at all times, and the participant must push up to a straight arm position. The test will be stopped when the participant strains forcibly or cannot maintain the appropriate technique within two repetitions. The outcome measure is the maximum number of push-ups performed consecutively without rest. An ICC of 0.83 was previously reported in college-age women performing bent-knee push-ups.^11^

#### Agility: Illinois Agility Run test

The length of the course will be 10 m, and the width will be 5 m. Four cones will be used to mark the start, finish and the two turning points. Another four cones will be placed down the centre an equal distance apart. Each cone in the centre will be spaced 3.3 m apart. The participant will be asked to lie on their front (head to the start line) with their hands by their shoulders. On the ‘Go’ command, the stopwatch will be started, and the participant must get up as quickly as possible and run around the course in the direction indicated, without knocking the cones over (or stepping over the cones), to the finish line, at which point the timing will be stopped. The total time in seconds will be recorded. The score will be the best of three attempts, and 1 min rest will be allowed between attempts. If improvements are made, participants may continue to perform the test beyond three attempts. Kutlu *et al*^12^ reported an ICC of 0.98 for repeated Illinois Agility Run test trials in amateur female football players. However, it should be noted that Kutlu *et al*^12^ used timing gates, a standing start and a minimum of 3 min rest between trials.

#### Muscular strength: handgrip

The participant will hold the dynamometer in their dominant hand, with their arm at a right angle, wrist slightly extending, and elbow by their side. The dynamometer handle will be adjusted if required—the base should rest on the first metacarpal (heel of palm), while the handle should rest on the middle of the four fingers. When ready, the participant will squeeze the dynamometer with maximum isometric effort, which will be maintained for about 5 s. No other body movement will be allowed. The participant will be strongly encouraged to give a maximum effort. The score is the best of three attempts; 1 min’s rest will be permitted between attempts. If improvements are made, participants may continue to perform the test beyond three attempts. Using an identical protocol, Gerodimos *et al*^13^ showed good reliability in 90 male basketball players, with an intraclass correlation (2,1) of 0.99 and a standard measurement error of 1.24 kg.

#### Power: countermovement jump

This test will be administered using the MyJump2 app, which calculates jump height by recording the jump and accurately selecting its take-off and landing. A smartphone or tablet will be positioned at the same height (1 m height) and the same distance (1.5 m) from the participant, enabling a clear view of the participant’s lower limbs. The sagittal plane is used to identify the exact take-off and landing frames more easily. Arm-swing will be prohibited; participants must keep their hands on the hips throughout the test. The test administrator will pay strict attention to the participant’s hands to ensure they are not used to apply additional force through the legs. Verbal instructions will be given to participants to rapidly descend by flexing at the knees and hips to approximately 90° and then immediately extend their knees and hips to ‘jump’ vertically up off the ground as high as possible, landing back on the mat on both feet at the same time. A 1-min rest will be allowed between trials. The take-off will occur from both feet simultaneously, with no initial steps or shuffling. Participants will not be allowed to pause at the bottom position of the squat. The best result of three attempts will be recorded. However, if improvements are made, participants may continue to jump beyond three attempts. It is also important that the athlete attempts to land in the same position as they took off, as jumping forwards, backwards, or sideways can affect the test results. To aid this, experimenters should stick tape to the floor as a marker for participants to take-off from and land on. The outcome measure is the highest jump recorded. Test-retest reliability of the CMJ using the My Jump app showed an ICC of 0.94 in recreationally active women.^14^

#### Flexibility: sit and reach

This test will involve sitting on the floor with legs stretched straight ahead. The soles of the participant’s bare feet will be placed flat against the box. Both knees will be locked and pressed flat to the floor; the tester may assist by holding them down. With the palms facing downwards and the hands on each other or side by side, the participant must reach forward along the measuring line as far as possible. Their hands must remain at the same level, not reaching further forward than the other. After some practice, they must reach out and hold that position for one to two seconds while the distance is recorded. There must not be any jerky movements. The score is the best of three attempts, and 1 min rest is allowed between attempts. If improvements are made, participants may continue to perform the test beyond three attempts. The outcome measure is the furthest score recorded. Jackson and Langford^15^ showed good reliability across two measurements in adult women (mean 35.1±7.5 cm, r^2^=0.99) using a similar protocol. The protocol must be standardised, as differences in muscle temperature and stretching exercises between testing phases will alter sit-and-reach distance. It is common for improvements in sit-and-reach scores to improve during the practice trials.

#### Incremental cycling test

Before and following the cycling protocol, a note will be made of any unique details of the test environment (*ie,* temperature, humidity, pressure), performance, or athlete concerns in the comments section of the data sheet. The incremental cycling tests will be performed on an electronically braked cycle ergometer, and oxygen uptake (V̇O_2_) and carbon dioxide output (V̇CO_2_) will be determined using open-circuit spirometry, open circuit spirometry and a breath-by-breath metabolic cart. Baseline heart rate (HR), blood lactate (La−) and blood pressure will be obtained before starting. All blood samples will be taken from the earlobe or finger; however, this must be consistent within a given research site. Participants will complete a 5-min warm-up at 45 W. The consumption of water is permitted during the warm-up.

*Part 1*: The protocol will encompass approximately five 4-min stages. The starting power output (W) will be amended for different training tiers/performance levels (see examples below). The familiarisation trials will help to determine the starting power output, which will be one of the following 15 W increments (ie, 45, 60, 75, 90, 105 W).

- Example 1: 45—warm-up; stage 1–45; stage 2–60; stage 3–75; stage 4–90; stage 5–105. This may not be enough to induce a blood lactate concentration of 4 mmol/L in some trained participants.
- Example 2: 45—warm-up; stage 1–90; stage 2–105; stage 3–120; stage 4–135; stage 5–150.

Participants can ride at a self-selected cadence, but not below 60 rev/min (recommend between 70 and 100 rev/min) and the cadence can vary up to 10% of the starting cadence during the test if desired. For each stage, power output, average HR, and rating of perceived exertion (RPE (0–10)) in the last 30 s, and blood [La−] values in the last 15 s will be recorded using a standardised spreadsheet. In sites where possible, blood pressure will be recorded for each increment within the last 30 s. Once a blood [La−] of ≥ 4 mmol/L is recorded, the protocol will be stopped, and the participant will receive a 5-min rest; the participant may remove the mouthpiece, and water may be consumed during this time, but they should remain seated on the cycle ergometer without pedalling. The last completed 4 min workload will be recorded as the maximal 4-min power output.

Part 2: the participant will be asked to begin pedalling at their preferred cadence against no load for 1 min. The test will begin at a one-step power output (ie, 15 W) higher than the last completed 4-min power output eliciting ≥4 mmol/L in part 1. After that, the load is increased by 15 W every 30 s until voluntary exhaustion. HR will be recorded at the end of each 30 s increment. Pedal cadence will be maintained within 10% of the participant’s preferred cadence established in part 1, and completion of the incremental cycling test will be signified by volitional cessation of exercise or an inability to maintain a pedal cadence. During the latter part of the test, the experimenter will verbally encourage the participants until voluntary termination. HR, power output, and RPE (RPE (0–10)) will be recorded at exhaustion. Participants will remain seated on the cycle ergometer for 3 min without pedalling, after which a single blood [La−] sample will be taken. Participants will then undertake a cool-down at a self-selected power output for 5 min.

A 7-breath rolling average will process all breath-by-breath files per Robergs and Burnett’s recommendations^16^ before data are extracted.

Part 1: 30-s averages for V̇O_2_ (mL/kg/min and L/min), V̇CO_2_, respiratory exchange ratio (RER), and HR will be determined for each 4-min stage. A third-order polynomial regression curve will represent the lactate response to exercise (power output and blood [La−]). These data will be used to determine Dmax, the exercise intensity that yields the maximum perpendicular distance from the third-order polynomial plotted curve from the lactate measures to the straight line between the first and the last data point,^17^ and onset blood [La−] accumulation at 4 mmol/L (OBLA^18^). Data will be analysed using The Lactate Thresholds App (ExPhysLab available at https://www.exphyslab.com/lactate/about, supported by R package lactater 0.1.1). Linear regression will then determine HR and V̇O_2_ at Dmax and OBLA. The outcome measures will be Dmax power output (W), blood [La−] and corresponding HR, and V̇O_2_, and OBLA 4 mmol/L power output, corresponding HR, and V̇O_2_.

Part 2: peak power output will be determined as the last stage a participant completes fully. If a participant finishes partway through a 30-s increment, peak power will be calculated as a percentage of the duration they completed the last power output. V̇O_2peak_ will be determined by averaging data in the last 30 s of the test. To ensure maximum effort, the following variables will also be reported at the end of the test (averaged 30 s RER *(criteria > 1.1), HR as a percentage of age-predicted maximum (Max HR_agepredicted_=220 age) (criteria > 95% HRmax), blood [La−] concentration (+3 min (criteria > 7 mmol/L)) and RPE (at exhaustion (criteria > 9/10)). The outcome measures will be V̇O_2peak_ (mL/kg/min and L/min), maximum HR and RER.

There is limited research in trained populations investigating the reliability of physiological variables obtained during incremental exercise tests comprising 4-min stages. However, from the limited literature available, the reliability of the OBLA is high (r=0.92) in trained women and men using an incremental running protocol comprising of 4-min work stages.^19^

### Hormone analysis

The same laboratory at the University of East Anglia will analyse all blood samples. Samples will be analysed using an electro-chemiluminescence immunoassay on a COBAS e601 analyser (Roche Diagnostics, Mannheim, Germany). The finding papers will report the actual interassay coefficient of variations (CV) and detection limits. Using the same laboratory and technique, Martin *et al*^20^ reported a CV for 17-β-oestradiol of <4.3% between 150 and 3000 pmol/L with a detection limit of 18.4–1581 pmol/L.

### Statistical considerations

Given the range of data collected and potential analyses, a clear distinction will be made between confirmatory and exploratory analyses. Confirmatory analyses will be preceded by a detailed analysis plan, which will be made publicly available, providing hypotheses to be tested along with statistical tests and relevant a priori thresholds. Hypotheses will be tested within groups assuming no difference (null hypothesis). Analyses will be conducted under the framework of multilevel models, with random intercepts included for both testing sites and individual participants. Multilevel models with repeated measurements nested in participants nested in sites will account for structure within the data and enable non-complete data to be analysed. A range of covariance structures to best model within-participant fluctuation over time will be explored. In addition to the analysis of means, variance parameters and their potential differences between groups will also be assessed. Exploratory analyses will primarily explore relationships across outcome domains, reducing dimensionality where possible and generating future hypotheses for subsequent data collection.

## Trial status

This study received ethical approval from seven sites worldwide: Australia, Brazil, Canada, Denmark, England, Finland and the USA. Data collection started in 2022 and is expected to conclude in 2024, and the results will be available after that. In addition to publishing the findings of this project, a process evaluation paper and a translational paper (ie, from paper to podium) will be produced, such that other researchers can learn from this multisite approach and athletes can learn from the findings of this laboratory-based study.

## Conclusions

Female athletes are under-represented and under-rated (*ie,* in terms of research quality) within sports and exercise science. This international, multisite, innovative project will address the lack of sufficiently powered, high-quality datasets in female athletes and has the potential to influence the development of female-specific guidelines in relation to the effects of MC and COCP phases on aspects of exercise physiology and athletic performance. In addition, the research design employed within this project has the potential to guide future high-quality studies in this research area.

## Data Availability

No data are available.
