# Supplementary material for: Effect of menstrual cycle and contraceptive pill phase on aspects of exercise physiology and athletic performance in female athletes: protocol for the Feminae international multisite innovative project
Source: BMJ Open Sport Exerc Med. 2023 Nov 24;9(4):e001814. doi: 10.1136/bmjsem-2023-001814 (PMC10679978; doi:10.1136/bmjsem-2023-001814)
Supplement: Supplementary data [file bmjsem-2023-001814supp001.pdf]

Training-related questions

Participant number	Which of these training phases are you currently in? Base training or Competition preperation or Competition season?	When do you expect to enter your next training phase? [answer in weeks/months from today - e.g., +2 weeks]	Will this change occur during the study timeframe? YES/NO	Which training phase [listed in column B] do you intend to enter next?	Year of birth?	Country of birth?	Country of residence?	Primary event as a senior athlete?	Secondary event as a senior athlete if applicable?	Self-assessment of highest level of achievement as senior athlete?	Years of dedicated training / competition?	How many hours of training do you do per week at the present time?	Will this change during the study timeframe? YES/NO	If yes, how many hours will you train for then?	When was your last off-season or break from training? [answer in weeks/months from today - e.g., -5 weeks]
1															
2															
3															
4															
5															
6															
7															
8															
9															
10															

Nutrition related questions

Participant number	General question: Do you deliberately and consistently follow a special/unique overall general dietary plan to support your training goals? YES/NO	If yes, which one? Vegan, vegetarian, Very high energy/extra energy (high Calorie), Restricted energy (low energy), Paleo, Paleo for athletes (Paleo but with more carbs around training sessions), Low Carb High Fat (LCHF), High protein Low Carb, Gluten Free, High Carb, Periodised Carb, FODMAP, Other - insert details [list all that apply]	Are the diets listed in column R your current diets? YES/NO	If no, which ones are your current ones?	When do you expect to enter your next dietary phase? [answer in weeks or months from today - e.g., +3 months]	Will this change occur during the study timeframe? YES/NO	Which dietary phase do you intend to enter next?	Do you use any supplements? YES/NO
1								
2								
3								
4								
5								
6								
7								
8								
9								
10								
